# A cross-sectional study: family communication, anxiety, and depression in adolescents: the mediating role of family violence and problematic internet use

**DOI:** 10.1186/s12889-023-16637-0

**Published:** 2023-09-07

**Authors:** Xin-cheng Huang, Yue-ning Zhang, Xiao-yu Wu, Yang Jiang, Hao Cai, Yu-qian Deng, Yuan Luo, Li-ping Zhao, Qin-ling Liu, Sheng-yue Luo, Yan-yan Wang, Li Zhao, Mao-min Jiang, Yi-bo Wu

**Affiliations:** 1 School of Economics and Management, Beijing Institute of Graphic Communication, Beijing, China; 2 School of Nursing, North Sichuan Medical College, Nanchong, China; 3 School of Pharmaceutical Science, Shandong University, Jinan, China; 4 Jitang College of North China University of Science and Technology, Tangshan, China; 5 Xiangya School of Public Health, Central South University, Changsha, China; 6 Xiangya School of Nursing, Central South University, Changsha, China; 7 The Second Xiangya Hospital of Central South University, Changsha, China; 8 School of Clinical Medicine, North Sichuan Medical College, Nanchong, China; 9 Department of Nuring, Affiliated Hospital of North Sichuan Medical Collge, Nanchong, China; 10 School of Public Affairs, Xiamen University, 422 Simingnan Road, Siming District, Xiamen, China; 11 School of Public Health, Peking University, Beijing, China

**Keywords:** Family relations, Anxiety, Depression, Internet use, Family violence, Adolescence

## Abstract

**Objective:**

The objective of this study is to explore the relationship between family communication, family violence, problematic internet use, anxiety, and depression and validate their potential mediating role.

**Methods:**

The study population consisted of Chinese adolescents aged 12 to 18 years, and a cross-sectional survey was conducted in 2022. Structural equation models were constructed using AMOS 25.0 software to examine the factors that influence adolescent anxiety and depression and the mediating effects of problematic internet use and family violence.

**Results:**

The results indicate that family communication was significantly and negatively related to family violence (*β* = -.494, *p* < 0.001), problematic internet use (*β* = -.056, *p* < .05), depression (*β* = -.076, *p* < .01), and anxiety (*β* = -.071, *p* < .05). And the finds also indicate that family violence mediated the relationships between family communication and depression (*β* = -.143, CI: -.198 -.080), and between family communication and anxiety (*β* = -.141; CI: -.198 -.074). Chain indirect effects between family communication and depression (*β* = -.051; CI: -.081 -.030) or anxiety (*β* = -.046; CI: -.080 -.043) via family violence and then through problematic internet use were also found in the present study.

**Conclusions:**

In conclusion, positive family communication is crucial in reducing anxiety and depression in adolescents. Moreover, problematic internet use and family violence mediate the effects of positive family communication on anxiety and depression. Therefore, improving family communication and promoting interventions aimed at reducing family violence and problematic internet use can help reduce anxiety and depression in adolescents, thus promoting their healthy development.

## Introduction

Adolescence is considered a crucial phase of individual development and transition, encompassing physiological, psychosocial, and cognitive transformations that often manifest as various psychological disorders [1]. Among these mental health issues, anxiety and depression have emerged as the most prevalent afflictions affecting adolescents [2]. Anxiety is frequently characterized by an excessive response to challenging or unpredictable situations, including obsessive–compulsive tendencies, worry, tension, and panic [3]. Conversely, depression represents a condition of persistent and pronounced sadness [4]. These two psychological disorders tend to exhibit similar traits and frequently co-occur, with approximately 85% of individuals with depression also experiencing significant anxiety symptoms, and 90% of those with anxiety disorders simultaneously presenting depressive symptoms [5]. Studies have revealed that globally, around 34% of adolescents aged 10–19 years are at risk of clinical depression, with a particularly elevated risk among Asian adolescents [6]. Another study, encompassing adolescent populations in 82 countries, indicated a global overall prevalence of anxiety among adolescents to be 9%. The persistence of depressive and anxious symptoms in adolescents can profoundly disrupt their developmental process and lead to considerable detriment in their social relationships and physical well-being [7]. Notably, during the Covid-19 pandemic, a quarter of adolescents worldwide experienced heightened depressive symptoms, while one-fifth grappled with significantly deeper anxiety symptoms. The pandemic exacerbated anxiety and depression in adolescents compared to previous reports from large-scale adolescent cohorts [8]. A meta-analysis conducted in China demonstrated that the Covid-19 pandemic notably impacted the mental health of over one-fifth of middle and high school students, revealing a higher prevalence of depressive and anxiety symptoms compared to studies not involving Covid-19 [9]. However, it is important to note that the prevalence of anxiety and depression varies significantly across countries. As a result, researchers have encouraged the adoption of socio-culturally specific strategies and tools, such as focusing on family relationships, to effectively prevent anxiety and depression problems [6].

The family, functioning as an ecosystem, assumes a highly significant role in the development of adolescent mental health by acting as a protective buffer against adverse mental health problems and reducing the risk of such issues arising [10]. From a sociological standpoint, family functioning profoundly influences the physical and mental well-being of individuals. Families that exhibit healthier functioning tend to offer greater emotional and material support to their members, assisting them in coping more effectively with negative conflicts [11]. Olson's Circumplex model of marital and family systems highlight family communication as the primary facilitating dimension for enhancing family cohesion and flexibility, both of which contribute significantly to the psychological well-being of family members [12]. Family communication, defined as the exchange of information, ideas, thoughts, and emotions among family members [13], serves as a pivotal bridge in transmitting emotions within the family and significantly influences the development of adolescent mental health [14]. Positive family communication is characterized by the ability of family members to comprehend, trust, and attentively listen to one another, while negative family communication typically involves an inability to engage in calm dialogues and express emotions and ideas effectively. León-Del-Barco and his colleagues underscored the pivotal role of the family environment in adolescent development, emphasizing that communication styles and behavioral patterns within the family exert a profound impact on adolescent mental health [15]. Moreover, family systems theory posits that problems among family members can often be traced back to miscommunication within the family unit [16]. Negative family communication, such as family conflict and reduced frequency of communication, can erode family relationships, lead to unhealthy family behaviors, and contribute to adolescent anxiety and depression [17]. Conversely, studies have demonstrated that positive parent-adolescent communication fosters a stronger bond between adolescents and their parents, promoting family cohesion and resilience, which serves as a crucial protective factor against adolescent depression [18]. Despite Geçer and Yıldırım's empirical study suggesting a direct impact of family communication in adults on psychological distress in the context of Covid-19 [19], very few studies have specifically investigated the influence of family communication on adolescents' psychological problems of anxiety and depression. Furthermore, there is a dearth of research exploring the mechanistic relationship between family communication and psychological disorders. In light of the Covid-19 impact, both parents and adolescents in China have been compelled to curtail their activities outside the home, resulting in prolonged close interactions with their family members [20]. In this context, the significance of family communication becomes even more prominent, and its impact on adolescents' mental well-being assumes greater importance. Therefore, this paper aims to explore the relationship between family communication and adolescents' anxiety and depression in the context of Covid-19, as well as the mechanisms that influence this relationship.

Adolescents exposed to family violence are at an increased risk of experiencing depression and anxiety. Family violence refers to the mental and physical abuse endured by an individual from other family members [21, 22]. Research has demonstrated that adolescents living in environments with parental family violence are more susceptible to depression compared to those not exposed to such family violence [23]. Furthermore, exposure to family violence not only heightens the likelihood of depression in adolescents but also leads to adverse physiological responses, such as elevated blood pressure, as a consequence of the depression [24]. Prolonged exposure to family violence during childhood, particularly from mothers, is associated with elevated psychological problems, such as depression and anxiety, during adolescence [25]. According to the Global Status Report on Violence Against Children 2020, jointly published by the World Health Organization and the United Nations Children's Fund, approximately one in two children between the ages of 2 and 17 years worldwide experiences some form of violence each year. Nearly 300 million children between the ages of 2 and 4 years are regularly subjected to violent discipline by their caregivers [26]. While authoritative statistics on family violence against adolescents in China are not available, data from reputable Chinese media indicates that almost 45% of parents have resorted to family violence against their children, primarily in the form of verbal abuse, and on average, a child experiences family violence once a month [27]. These statistics underscore the frequency of adolescents' exposure to family violence. The Behavioral Family Systems Model posits that communication skills play a vital role in family functioning, reducing family conflict, promoting positive family relationships, and enhancing adolescents' resilience in coping with stress [28]. Olson's Circumplex model of marital and family systems has consistently revealed that family communication fosters family cohesion and resilience, as supported by a wealth of empirical studies [29–31]. Furthermore, high levels of family cohesion and resilience have been associated with decreased exposure to family abuse [32]. Therefore, it is reasonable to infer that family communication acts as a protective factor for adolescents against family violence and, consequently, alleviates psychological problems such as anxiety and depression. Especially during the Covid-19 pandemic, when family members have had more frequent interactions, the potential for the occurrence of family violence has increased [33], subsequently elevating the likelihood of adolescents experiencing anxiety and depression [34]. More explicitly, we can hypothesize that family communication's mitigating effect on anxiety and depression in adolescents is achieved through its role in reducing family violence.

Problematic Internet use emerges as a significant contributing factor to the heightened risk of depression and anxiety in adolescents. This issue shares behavioral characteristics akin to drug addiction, alcoholism, and gambling addiction, and exerts adverse effects on an individual's social relationships, as well as their physical and mental well-being [35]. Numerous studies have substantiated the association between problematic Internet use and adverse mental health outcomes. For instance, a cross-sectional study by Li et al. demonstrated a direct increase in the risk of depression among Chinese middle school students due to problematic Internet use [36]. Adolescents exhibiting problematic Internet use displayed significantly poorer mental and emotional states compared to their counterparts without such behaviors [37]. Particularly during the Covid-19 pandemic, the correlation between problematic Internet use and adolescent anxiety and depressive symptoms became evident, with related studies highlighting the direct impact of problematic Internet use on anxiety and depression [38]. The detrimental impact of problematic Internet use on adolescent mental health can be attributed to the tendency for excessive Internet engagement to reduce real-world social interactions among adolescents [39]. As they spend more time in the virtual world, they may have less interaction with their friends and family in real life. This substitution of "virtual socialization" for "real socialization" may exacerbate symptoms of anxiety and depression. However, it is noteworthy that Chinese adolescents' Internet use is often regulated by their parents, making the family a crucial influence on adolescents' problematic Internet use. Nielsen, Favez, and Rigter's literature review on parenting, family factors, and adolescents' problematic Internet use highlighted that positive family parenting and family dynamics were associated with lower problematic Internet use, while negative parenting and family dynamics were linked to higher rates of problematic Internet use [40]. When parents maintain a positive communication relationship with their children, such as actively listening and understanding their thoughts, it typically reduces adolescents' problematic Internet use [41]. Similarly, positive family environments, such as increased family expression, have been shown to reduce adolescents' problematic Internet use and time spent online [42]. Family systems theory posits that individual and family members' behaviors are influenced by the patterns of family relationships, which can have an intergenerational transfer effect [43]. For instance, individuals in families with healthier communication patterns are more likely to develop healthy interpersonal relationship patterns in the future. Conversely, unhealthy communication within the family may lead to problematic interpersonal relationships or even conflict and violence in the future. In summary, we propose that adolescents' problematic Internet use likely serves as a mediator of the effect of family communication on their anxiety and depression. Positive family communication can help reduce adolescents' problematic Internet use, consequently lowering their risk of anxiety and depression. Furthermore, when family communication is positive, there is a corresponding reduction in family conflict and violence, which may also play a significant role in reducing adolescents' problematic Internet use.

**Fig. 1:**
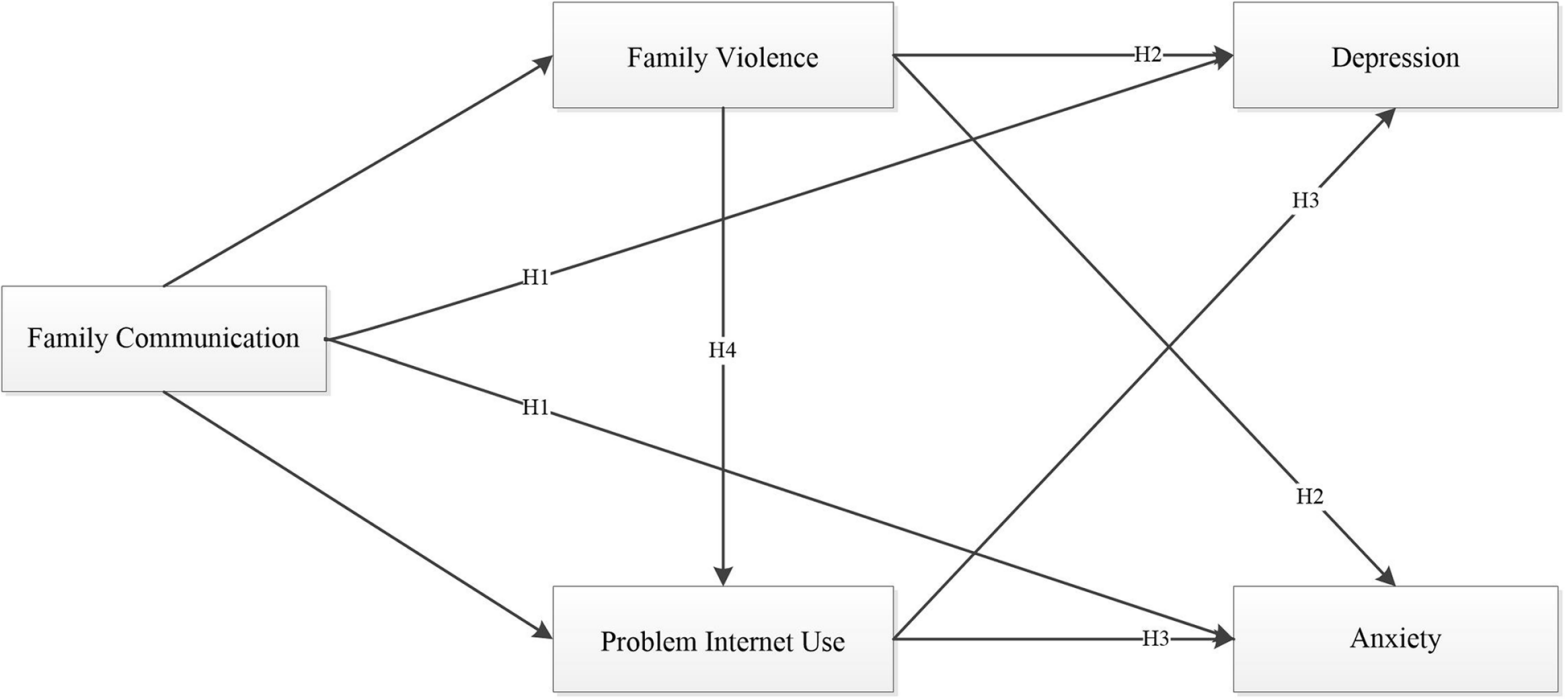
Research hypotheses model diagram

In this paper, we aim to investigate the relationship between adolescent family communication and anxiety and depression, as well as explore the potential mediating roles of family violence and problematic Internet use. We propose the following research hypotheses and present the research model diagram (see Fig. 1):

- Hypothesis 1: Family communication is negatively associated with adolescent anxiety and depression.
- Hypothesis 2: Family violence mediates the relationship between family communication and adolescent anxiety and depression.
- Hypothesis 3: Problematic Internet use mediates the relationship between family communication and adolescent anxiety and depression.
- Hypothesis 4: Family violence and problematic Internet use jointly play a chain mediating role between family communication and adolescent anxiety and depression.

## Methods

### Participants and procedure

**Fig. 2:**
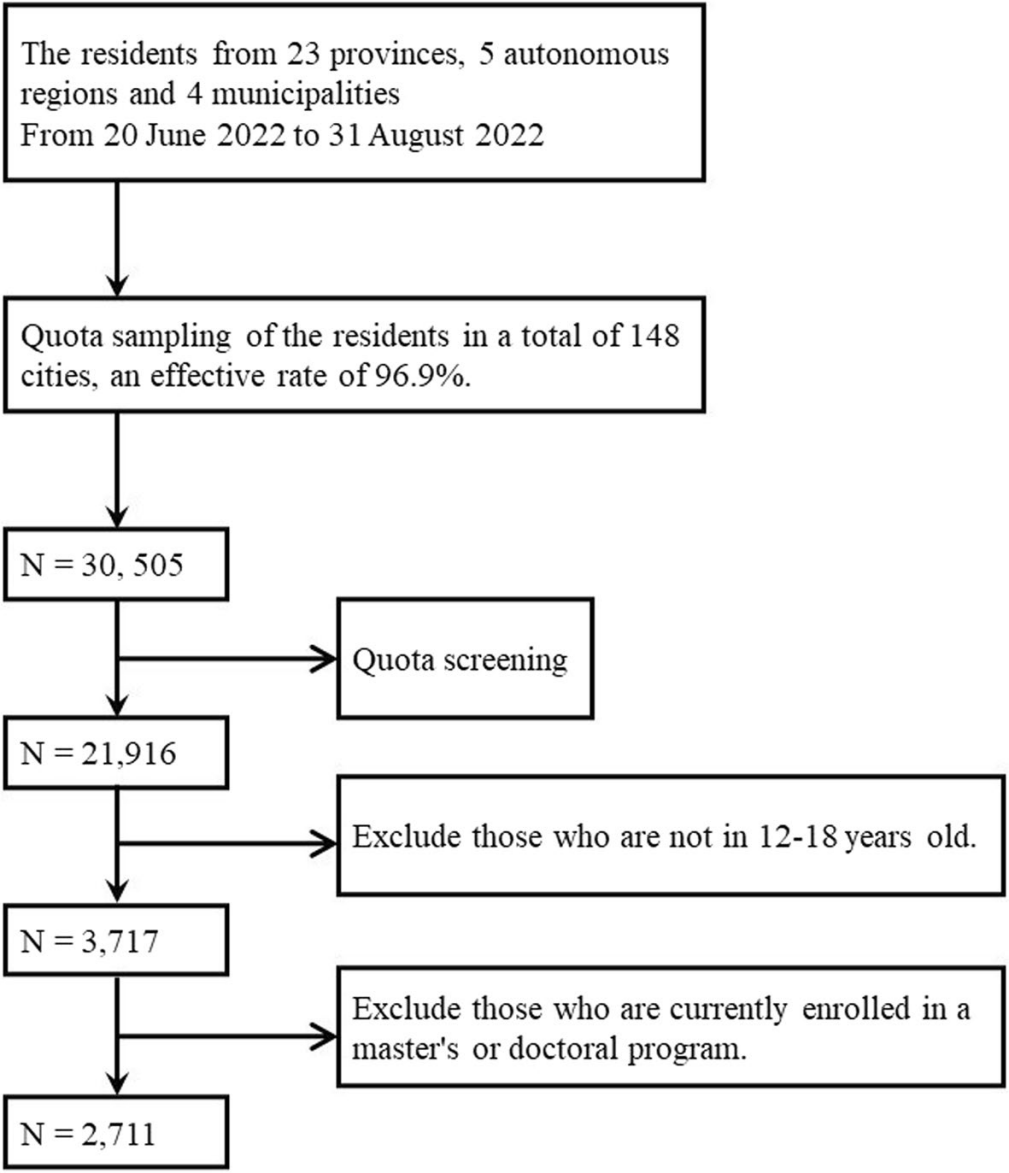
Sample screening process

The data used in this study comes from the "2022 China Population Psychological and Behavioral Survey". The survey was carried out from June 20 to August 31, 2022, in 148 cities, 202 counties, 390 towns/streets, and 780 communities/villages (excluding Hong Kong, Macao, and Taiwan) across 23 provinces, 5 autonomous regions, and 4 municipalities directly under the central government in China [44]. A large number of investigators were recruited and rigorously trained nationwide for the survey. Respondents were invited to respond face-to-face and one-on-one through an electronic questionnaire. Participants will be asked to sign an informed consent form before responding. A total of 31,480 questionnaires were distributed, and 30,505 valid questionnaires were finally collected, resulting in an effective rate of 96.9%. For this study, adolescents aged 12–18 years were selected from the data, and after excluding the samples with illogical answers such as who are currently enrolled in a master's or doctoral program, a total of 2,711 valid samples were obtained. The sample screening process is shown in Fig. 2. This study has been officially registered with the China Clinical Trials Registry (Registration number: ChiCTR2200061046, 15/06/2022) and has been approved by the Clinical Research Ethics Committee of the Second Xiangya Hospital of Central South University (No.2022-K050).

Of all 2,711 samples, 1,601 (59.1%) were female, 1,742 (64.3%) were high school and below, 1,960 (72.3%) lived in urban areas, and 438 (16.2%) had a per capita monthly household income of more than 9,000.

### Measures

#### General characteristics

The general characteristics of the participants were collected, including gender, age, residence, education level, and monthly per capita household income.

#### Family communication

The assessment of family communication was carried out using the Family Communication Scale (FCS) developed by Olson et al. (2011). This scale consists of 10 items designed to evaluate the quality of communication among family members from the individual's perspective, as well as the communication skills and abilities of family members. Sample items include "Family members are satisfied with the way they communicate with each other," "Family members are able to have calm discussions about issues," and "Family members receive honest responses when they ask each other questions." Participants rated their responses on a five-point Likert scale, where 1 indicates strong disagreement and 5 represents strong agreement (Cronbach's α = 0.969; M = 36.64, SD = 8.94). A higher total score indicates a higher quality of family communication. During the data analysis phase, we randomly divided the 10 questionnaire items into 3 factors to enhance the model fit.

#### Family violence

To measure the violence experienced by adolescents from family members, a five-item family violence scale was developed, comprising two dimensions: mental violence and physical violence. (a) The mental violence dimension includes three items: "My family does not care about me when I am in a bad state (physically unwell or in a bad mood)." "My family invades my privacy by going through my cell phone, dictates what I wear, and restricts my interpersonal interactions." "My family compares me to others and openly accuses me, causing me embarrassment and undermining my self-confidence." (b) The physical violence dimension consists of two items: "My family has physically harmed me using direct blows or objects." "My family has engaged in unwanted physical contact with me." Participants rated their responses on a five-point Likert scale, where 1 indicates "almost never," 2 denotes "sometimes," and 3 stands for "often" (Cronbach's α = 0.901; M = 7.70, SD = 4.04). The total score was calculated by summing the scores of the five question items, with higher scores indicating a higher level of exposure to family violence. In this study, the Cronbach's α of the Family Violence Scale was 0.901, indicating good internal consistency. Moreover, the results of the confirmatory factor analysis demonstrated that the scale exhibited high reliability, as evidenced by the following fit indices: Comparative Fit Index (CFI) = 0.932, Normed Fit Index (NFI) = 0.931, Goodness of Fit Index (GFI) = 0.910, Incremental Fit Index (IFI) = 0.932, and Standardized Root Mean Square Residual (SRMR) = 0.046.

#### Problematic internet use

This study employed the Problematic Internet Use Questionnaire Short Form (PIUQ-SF-6) developed by Opakunle et al. [45] to measure problematic Internet use. The scale comprises three dimensions: obsession, neglect, and control, with a total of six question items, two for each dimension. For instance: "Have you ever experienced feelings of worry, moodiness, or tension when not online, but once you are online, these feelings disappear?" "Do you feel nervous, irritable, or stressed if you are unable to go online when you want to?" Participants provided responses to each question item using a 5-point Likert scale, ranging from "1 = never" to "5 = all the time" (Cronbach's α = 0.911; M = 14.23, SD = 5.62). Higher scores on the scale indicate a higher degree of problematic Internet use, suggesting a stronger association with Internet-related issues and potential negative consequences.

#### Anxiety

The 7-item Generalized Anxiety Disorder Scale (GAD-7) was developed by Spitzer et al. [46] and has gained widespread usage in clinical settings and various studies, particularly for screening anxiety disorders. The GAD-7 comprises seven items that assess symptoms of generalized anxiety, such as "feeling tense, anxious, or on edge" and "not being able to stop or control worrying." Participants were asked to rate how often they experienced these symptoms in the past two weeks based on their feelings. The scale was rated on a four-point scale, with responses ranging from "0 = not at all" to "3 = almost every day" (Cronbach's α = 0.954; M = 12.33, SD = 5.24). Higher scores on the scale indicate a higher level of anxiety severity. During the data analysis phase, the 7 question items of the scale were randomly divided into 3 factors to enhance the fit of the model, making it more suitable for the study's purposes.

#### Depression

Depression was measured using the well-established 9-item Depression Screening Scale known as the Patient Health Questionnaire (PHQ-9). This self-rating scale is a simple yet effective tool for screening individuals for depression and is widely used internationally, with translations available in multiple languages [47, 48]. The PHQ-9 comprises nine common symptoms associated with depression, such as "feeling down, depressed, or hopeless" and "having little interest or pleasure in doing things." Participants were asked to rate how often they experienced these symptoms in the past two weeks based on their feelings. Responses were recorded on a four-point scale, with options ranging from "0 = not at all" to "3 = almost every day" (Cronbach's α = 0.928; M = 16.40, SD = 6.19). Higher scores on the scale indicate a higher level of depression severity. During the data analysis phase, the 9 question items of the scale were randomly grouped into 3 factors to enhance the model fit, making it more suitable for the study's analytical purposes.

### Statistical analysis

The data were analyzed using SPSS 25.0 and AMOS 25.0, with two-tailed p-values at the 0.05 level considered statistically significant. Descriptive statistics, such as mean, standard deviation, and sample size, were computed using SPSS 25.0. Scale reliability and validity tests, path analysis, and mediation tests were conducted using AMOS 25.0. To test the hypothesized mediation models, a structural equation model (SEM) with asymptotically distribution-free estimation was employed. The analysis assessed direct, indirect, and total effects through 2,000 bootstrapped samples to ensure robustness and accuracy of the results. Effect estimates and bias-corrected 95% confidence intervals (CIs) were calculated to provide a comprehensive understanding of the relationships between the variables under investigation.

## Results

### Scale reliability and validity tests

#### Reliability and convergent validity tests of scales

In this paper, all dimensions of all scales were analyzed by CFA and the results showed that all factor loadings met the criteria recommended by Joreskog and Sorbom of greater than 0.45 and significant [49]. Further, the component reliabilities were all greater than or equal to 0.7, while the mean variance extractions ranged from 0.906 to 0.968 (Table 1), which meets the discriminant criteria proposed by Fornell and Larcker [50] and Chin [51], i.e., the Composite Reliability (CR) value should be greater than 0.7, the AVE value should be no less than 0.5, and the factor loadings for each question item should be greater than 0.5. Therefore, the five scales presented high reliability and convergent validity.

**Table 1:** CFA factor analysis and convergent validity

Construct	Item	Significance of Estimated Parameter				Item Reliability		Composite Reliability	Convergence Reliability
		Unstd	S.E	Z-value	*P*	Std	SMC	CR	AVE
Family Communication	FC1	1.000				.973	.947	.968	.911
	FC2	.989	.008	117.911	^***^	.946	.895		
	FC3	1.009	.009	116.969	^***^	.944	.891		
Family Violence	FV1	1.000				.876	.767	.906	.660
	FV2	.919	.016	57.964	^***^	.858	.736		
	FV3	1.063	.023	46.707	^***^	.751	.564		
	FV4	1.046	.019	53.828	^***^	.820	.672		
	FV5	1.090	.023	46.453	^***^	.748	.560		
Problematic Internet Use	PIU1	1.000				.753	.567	.911	.632
	PIU2	1.090	.024	44.797	^***^	.839	.704		
	PIU3	1.136	.026	43.541	^***^	.817	.667		
	PIU4	1.032	.025	40.535	^***^	.767	.588		
	PIU5	1.089	.027	40.540	^***^	.767	.588		
	PIU6	1.099	.025	43.755	^***^	.821	.674		
Depression	DE1	1.000				.916	.839	.936	.829
	DE2	1.103	.015	75.485	^***^	.915	.837		
	DE3	1.002	.014	73.066	^***^	.901	.812		
Anxiety	AN1	1.000				.936	.876	.953	.871
	AN2	1.058	.012	90.463	^***^	.935	.874		
	AN3	1.013	.011	88.529	^***^	.928	.861		

^***^ *P* < 0.001

#### Reliability and convergent validity tests of scales

In this study, the AVE method proposed by Fornell and Larcker was used to test the discriminant validity of the variables [50]. The AVE method means that the average variance extracted for each dimension must be greater than the squared value of the correlation coefficients between the dimensions and the dimensions, but since the AVE is a squared value, it must be converted into the same squared unit if it is to be compared with the Pearson correlation between the dimensions. Therefore, the AVE value is generally rooted and then compared, and if it is higher than the value of the inter-dimension Pearson correlation, the dimension can be claimed to have differential validity. In this study (Table 2), the AVE values are in bold diagonal, and all AVE values are greater than the standardized correlation coefficients under the diagonal. Therefore, we consider the validity of the distinction between the study variables to be good.

**Table 2:** Analysis of AVE distinguishing validity

Construct	AVE	(1)	(2)	(3)	(4)	(5)
(1) Anxiety	.871	**.933**				
(2) Depression	.829	.868	**.910**			
(3) Problematic Internet Use	.632	.450	.448	**.795**		
(4) Family Violence	.660	.449	.448	.428	**.812**	
(5) Family Communication	.911	-.291	-.300	-.251	-.493	**.954**

Square root of AVE in bold on diagonals. Off diagonals are Pearson correlation of constructs

### Overall model fit analysis

This study uses the model fitting criterion recommended by Hu and Bentler [52], including *χ*^*2*^*/df* < 3, Goodness of Fit ≥ 0.95, Adjust Goodness of Fit ≥ 0.95, Tucker Lewis Index (TLI) of ≥ 0.95, Comparative Fit Index (CFI), Normed Fit Index (NFI) ≥ 0.95, Root Mean Square Error of Approximation (RMSEA) of ≤ 0.08 and Standardized Root Mean Square Residual (SRMR) of ≤ 0.08.

First of all, since the dependent variables Depression and Anxiety have strong correlation, while their relationships and difference are not the focus of this study. Therefore, we established a correlation between them in the model fitting stage in order to improve the overall model fitting effect. Secondly, when the sample size is larger than 1,000 and does not conform to a positive-trait distribution, Browne's recommendation of asymptotically distribution-free model estimation can be used [53]. Thirdly, since the sample size of this study reaches 2711, the model chi-square value will result in a significant p-value due to the large sample size and make the sample fit poorly to the model matrix. Therefore, we used the bootstraps method proposed by Bollen and Stine to correct the model fit indicators [54]. The chi-square value obtained after the Bollen-Stine P correction analysis was 184.62, while the original model had a chi-square value of 754.53, so all other fitness metrics needed to be recalculated. Then, the calculated fitness metrics show that the model of this study has a good fit, indicating that the model of adolescent family communication and mental health constructed by the sample of this study can be used to explain the actual observed data, and the fit results are presented in Table 3.

**Table 3:** Bollen-Stine P Correction model fit metrics

Fit metrics	Acceptable ranges	The present model fits	Results
*χ*^*2*^	the smaller the better	184.62	Good
*χ*^*2*^*/df*	≤ 3.00	1.15	Accepted
GFI	≥ .95	.96	Accepted
AGFI	≥ .95	.95	Accepted
TLI	≥ .95	.99	Accepted
CFI	≥ .95	.99	Accepted
NFI	≥ .95	.96	Accepted
RMSEA	< .08	.01	Accepted
SRMR	< .08	.07	Accepted

SRMR was not rectified by the Bollen-Stine P correction method

### Path testing for structural equation model

**Fig. 3:**
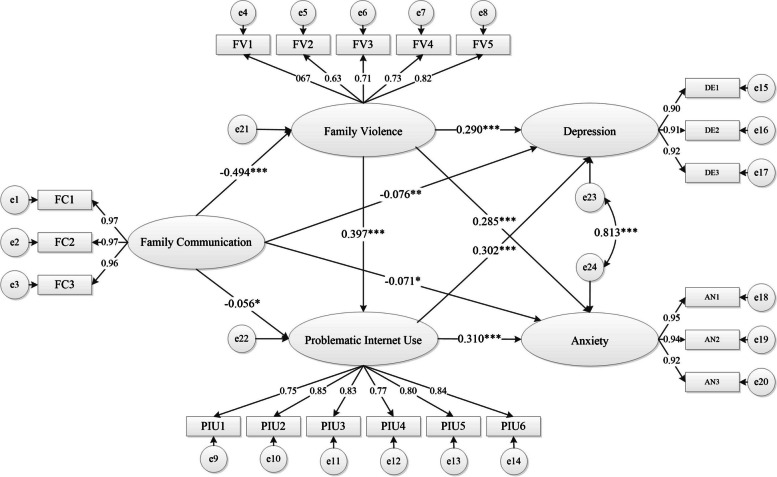
The structural equation model for mediations among family communication, depression and anxiety

Regarding the path analysis for the structural equation model, family communication was significantly and negatively related to family violence (*β* = -0.494, *p* < 0.001), problematic internet use (*β* = -0.056, p < 0.05), depression (*β* = -0.076, *p* < 0.01), and anxiety (*β* = -0.071, *p* < 0.05) with moderate or small effect sizes. In contrast, family violence exhibited significant and positive relations with problematic internet use (*β* = 0.397, *p* < 0.001), depression (*β* = 0.290, *p* < 0.001), and anxiety (*β* = 0.285, *p* < 0.001) with moderate or small effect sizes. Furthermore, problematic internet use was significantly and positively related to depression (*β* = 0.302, *p* < 0.001) and anxiety (*β* = 0.310, *p* < 0.001) with moderate effect sizes. The results were presented in Table 4 and Fig. 3.

**Table 4:** Path results of the research model

Paths	Unstd	Std	S.E	T	*P*
FC → FV	-.201	-.494	.014	-13.946	***
FC → PIU	-.058	-.056	.027	-2.127	.033
FC → DE	-.052	-.076	.020	-2.665	.008
FC → AN	-.054	-.071	.021	-2.546	.011
FV → DE	.492	.290	.068	7.259	***
FV → AN	.536	.285	.073	7.298	***
PIU → DE	.203	.302	.017	12.074	***
PIU → AN	.230	.310	.018	12.768	***
FV → PIU	1.003	.285	.084	12.002	***

*FC* Family Communication, *FV* Family Violence, *PIU* Problematic Internet Use, *DE* Depression, *AN* Anxiety

^***^ *P* < 0.001

### Mediating role analysis

Indirect effects among family communication, depression and anxiety were analyzed by performing a bootstrap with 2000 resamples and a bias-corrected and Percentile confidence interval of 95%. As is shown in Table 5, four indirect effects were significant among family communication, depression and anxiety. Family violence mediated the relationships between family communication and depression (*β* = -0.143, CI: -0.198 -0.080), and between family communication and anxiety (*β* = -0.141; CI: -0.198 -0.074). Chain indirect effects between family communication and depression (*β* = -0.051; CI: -0.081 -0.030) or anxiety (*β* = -0.046; CI: -0.080 -0.043) via family violence and then through problematic internet use were also found in the present study. Besides, the proportional values of indirect-only mediated effects of the four is 65.30%, 66.51%, 38.64%, and 42.42%. The model explained 46.21% of the participants anxiety and 33.6% of the participants depression. However, the mediating role of problematic internet use in the relationship between family communication and anxiety and depression was not significant. As a result, H4 was supported, while H3 was not.

**Table 5:** Path indicators and proportions of mediating effects between family communication, depression and anxiety

Paths	T. E	D. E	I. E	S. E	P. E	Bias 95%		Percentile 95%	
						LL	UL	LL	UL
FC-FV-DE	-.219	-.076^a^	-.143	.028	65.30%	-.198	-.080	-.194	-.078
FC-FV-AN	-.212	-.071^a^	-.141	.029	66.51%	-.198	-.074	-.187	-.064
FC-FV-PIU-DE	-.132	-.076^a^	-.056	.012	42.42%	-.081	-.030	-.078	-.028
FC-FV-PIU-AN	-.132	-.071^a^	-.061	.009	46.21%	-.080	-.043	-.076	-.040

Only an indirect path with Empirical 95% confidence interval is presented, and it doesn’t overlap with zero; Bootstrap sample size = 2000; ^a^ represent statistically significant at 1‰ level. *S. E.* Standard errors, *T. E.* Total effects, *D. E.* Direct effects, *I. E.* Indirect effects, *P. E.* Proportional values of indirect-only mediated effects, *FC* Family Communication, *FV* Family Violence, *PIU* Problematic Internet Use, *DE* Depression, *AN* Anxiety, *LL* Lower Limit, *UL* Upper Limit

## Discussion

This study focused on anxiety and depression among Chinese adolescents during the Covid-19 context and utilized structural equation modeling to examine the relationship between family communication and adolescents' anxiety and depression. Additionally, the study explored the potential mediating roles of family violence and problematic Internet use in this context. The results of the study revealed a significant negative correlation between family communication and adolescents' anxiety and depression, indicating that positive family communication can effectively reduce adolescents' psychological distress. Moreover, family violence was found to mediate the relationship between family communication and anxiety and depression, suggesting that family violence may play a crucial role in influencing the association between family communication and adolescent mental health. Contrary to Hypothesis H3, problematic Internet use did not mediate the relationship between family communication and anxiety and depression. However, the study confirmed the presence of a common chain mediating role involving both family violence and problematic Internet use, linking them to the impact of family communication on adolescent anxiety and depression. Overall, the findings of the study support hypotheses H1, H2, and H4, and provide valuable insights into the factors influencing adolescent mental health problems. These findings have significant implications for understanding the role of family communication and violence in adolescents' mental well-being, offering useful directions for family communication and violence interventions aimed at improving the mental health of adolescents.

### Positive family communication can reduce the risk of anxiety and depression in adolescents

Based on the results of this study, it was found that positive family communication plays a significant role in reducing the risk of anxiety and depression in adolescents. This suggests that adolescents who experience a more positive and open family communication environment are less likely to suffer from severe anxiety and depression. These findings align with a study conducted by Zhou et al. [55], which also demonstrated that adopting positive and conversation-oriented family communication can prevent adolescents' risk of depression. Bowen's family systems theory proposes that each family operates within an emotional system where emotional connections among family members are interconnected. Positive family communication involves the free flow of information, empathic understanding between parents and children, clear and accurate messaging, consistent parenting, and satisfying interactions [56]. Such positive communication fosters a sense of security, controllability, and predictability in adolescents' lives, leading to a reduction in negative emotions [57, 58]. On the other hand, unhealthy family communication, characterized by negativity and avoidance, can contribute to increased stress in adolescents' school and personal lives, thereby increasing their vulnerability to anxiety and depression [57]. It is essential to highlight the significance of family communication, especially during the Covid-19 pandemic, when adolescents are exposed to vast amounts of information related to illness and risk. These changes in information processing and learning patterns can easily impact their mental health, leading to issues like anxiety and depression [59]. Family communication becomes a crucial means of coping with uncertainty during such challenging times. Active communication with parents allows adolescents to obtain accurate information about the epidemic, reducing their perception of future uncertainty and risk, and alleviating adverse emotions [60]. As a result, family interventions targeted at adolescents' anxiety and depression should prioritize improving the family communication climate. Parents should learn to listen to their children's thoughts and reduce critical and blaming attitudes. The findings of this study underscore the importance of positive family communication in promoting adolescent mental health. By promoting such communication, we can effectively reduce the risk of anxiety and depression in adolescents, especially during challenging situations like the Covid-19 pandemic. Future family interventions should focus on enhancing the family communication environment to aid adolescents in better coping with stress and emotional challenges.

### Family violence mediates the relationship between family communication and anxiety and depression

Another significant research discovery pertains to the mediating role of family violence between family communication and adolescents' anxiety and depression. This suggests that fostering positive family communication may assist adolescents in avoiding victimization from family members, thereby reducing their susceptibility to anxiety and depression. Jiménez's study [57] demonstrated that family communication problems serve as a risk factor for family violence, while open communication aids in mitigating this risk. The Circumplex Model framework of family functioning proposed by Olson et al. [61] helps elucidate this relationship. Family communication plays a pivotal role in promoting family cohesion and flexibility. Families facing communication challenges are more likely to exhibit low flexibility (i.e., difficulty in adapting norms and rigidity) and low cohesion (i.e., inadequate connections among family members). In essence, positive family communication fosters greater family cohesion and flexibility, serving as a protective factor against adolescents falling prey to family violence, thus promoting their mental well-being. Positive family communication places emphasis on harmonious dialogue among family members, whereas inadequate family communication tends to be submissive and contradictory [62]. Conversation-oriented family communication typically enhances adolescents' mental health, whereas conformity-oriented family communication, conversely, exhibits a negative correlation with adolescents' mental well-being [63]. In China, family education often follows an authoritative approach, leading to a higher likelihood of adolescents becoming victims of family violence, especially from parents [64]. Authoritarian family upbringing usually involves less emotional support and more criticism and behavioral control [65], resulting in strained parent–child relationships and an increased risk of depression [66]. The context of Covid-19 has significantly increased the occurrence of family violence [67]. This is due to the elevated risk factors brought about by Covid-19, resulting in heightened uncertainty among family members and a greater propensity for conflicts. Moreover, with most family members living in close quarters due to Covid-19, parental emotions from work or daily life may be displaced onto children or adolescents. Consequently, harmonious communication, mutual understanding, and effective problem-solving within the family have been recognized as crucial clinical strategies for mitigating family conflict and violence stemming from Covid-19 [68]. These findings provide valuable insights into the influence of family communication and family violence on adolescent mental health. In practice, there should be a strong emphasis on promoting positive family communication, alongside efforts to strengthen family violence intervention and prevention measures. Particularly in the context of Covid-19, it is imperative to implement effective measures to ameliorate the adverse effects of family conflict and violence and safeguard the mental well-being of adolescents.

### Family violence and problematic internet use as chain mediators between family communication and the relationship between anxiety and depression

The findings suggest that while problematic internet use does not directly act as a mediating mechanism between family communication and anxiety and depression, family violence and problematic internet use function as co-chain mediators in this relationship. This novel contribution to existing literature highlights the role of problematic internet use as a consequence of family violence, which, in turn, influences adolescent anxiety and depression. Specifically, we observed that inadequate family communication may result in mental or physical violence from family members, leading to an increase in problematic internet use among adolescents, ultimately exacerbating their mental health issues. This phenomenon can be elucidated through the lenses of self-regulation theory and approach-avoidance theory. According to self-regulation theory, individuals tend to employ specific strategies to regulate and manage their emotional and psychological states [69]. When adolescents face violence from family members, they may lack healthy and positive emotion regulation strategies, such as seeking social support, expressing emotions, or actively problem-solving. Consequently, problematic internet use becomes a seemingly simple and instant escape, enabling them to temporarily alleviate their discomfort without addressing the underlying problem. Additionally, approach-avoidance theory suggests that individuals may respond in an avoidance or coping manner when confronted with stressful events. Given that the adolescent population is mentally immature and possesses limited coping skills, they may be more inclined to adopt an avoidance response [70]. In the context of Covid-19, external avoidance approaches may be less accessible to adolescents, leading them to turn to the Internet as their primary means of venting and regulating their internal emotions. Nevertheless, this escape may further intensify their negative psychological emotions [38]. However, it is essential to acknowledge that the relationship between adolescents' family relationships and mental health is complex and contradictory, necessitating further research to delve deeper into how family relationships, family violence, and problematic internet use impact adolescents' mental well-being. Particularly in the context of Covid-19, changes in family and online environments may have a more intricate influence on adolescents' mental health, calling for more in-depth exploration and understanding of this domain.

### Limitations and implications

The study's limitations warrant consideration. Firstly, the cross-sectional design employed restricts the establishment of causal relationships between family communication and anxiety and depression. Future research should adopt a longitudinal design to ascertain the directionality of these associations. Secondly, the study solely focused on family communication as a family factor; to gain a more comprehensive understanding, future research should incorporate other factors like family satisfaction, parental mental health, and family socioeconomic status. Thirdly, the study exclusively included adolescents, necessitating future research to encompass a broader sample group to better comprehend the impact of family violence. Lastly, the study solely concentrated on family violence and problematic internet use as mediators, overlooking other potential mechanisms. Future research should explore additional social, school, family, and individual factors beyond the study's scope.

Despite these limitations, the study's results carry significant practical implications. It stands as one of the few inquiries delving into the mechanisms underlying the relationship between family communication, family violence, problematic internet use, anxiety, and depression. The findings underscore the significance of family communication and family violence in influencing problematic behaviors and psychological well-being among adolescents, emphasizing the necessity to enhance adolescents' communication skills with their parents and reduce the incidence of family violence. Furthermore, while previous studies have mainly investigated the effects of family relationships on perpetrators, this study fills an important gap by shedding light on the psychological and behavioral manifestations experienced by adolescent victims of family violence from their perspective.

## Conclusion

This study sheds light on the connections between family communication, family violence, and problematic internet use in relation to adolescent anxiety and depression, providing valuable insights into their underlying mechanisms. Particularly, positive family communication exhibits a negative association with adolescent anxiety and depression, indicating that fostering healthy family communication can mitigate negative psychological symptoms in adolescents. Furthermore, family violence acts as a mediator between family communication and anxiety and depression, underscoring the potential of improving family communication to prevent adolescents from becoming victims of family violence and indirectly reduce their vulnerability to anxiety and depression. Additionally, the study reveals a co-chain-mediated effect of family violence and problematic internet use on the relationship between family communication and anxiety and depression, suggesting that adolescents may employ problematic internet use as a coping mechanism for dealing with the negative emotions stemming from family violence. Consequently, enhancing family communication skills emerges as a significant approach to alleviate adolescents' anxiety and depression, consequently reducing the prevalence of family violence and problematic internet use.

These findings carry significant implications for families, educational institutions, and society at large. In the endeavor to help adolescents alleviate anxiety and depressive symptoms, a strong emphasis should be placed on the importance of family communication, promoting understanding and support among family members through positive communication. Concurrently, proactive measures should be taken to prevent and intervene in family violence, safeguarding the physical and mental well-being of adolescents. Moreover, attention should be given to adolescents' problematic internet use, guiding them in the proper utilization of internet resources and discouraging excessive reliance on virtual socialization, as this can contribute to a reduction in their anxiety and depression levels.

## Data Availability

Data are available, upon reasonable request, by emailing: bjmuwuyibo@outlook.com.
