# Correction: A cross-sectional study: family communication, anxiety, and depression in adolescents: the mediating role of family violence and problematic internet use

**DOI:** 10.1186/s12889-023-17226-x

**Published:** 2023-11-27

**Authors:** Xin-cheng Huang, Yue-ning Zhang, Xiao-yu Wu, Yang Jiang, Hao Cai, Yu-qian Deng, Yuan Luo, Li-ping Zhao, Qin-ling Liu, Sheng-yue Luo, Yan-yan Wang, Li Zhao, Mao-min Jiang, Yi-bo Wu

**Affiliations:** 1 School of Economics and Management, Beijing Institute of Graphic Communication, Beijing, China; 2 School of Nursing, North Sichuan Medical College, Nanchong, China; 3 School of Pharmaceutical Science, Shandong University, Jinan, China; 4 Jitang College of North China University of Science and Technology, Tangshan, China; 5 Xiangya School of Public Health, Central South University, Changsha, China; 6 Xiangya School of Nursing, Central South University, Changsha, China; 7 The Second Xiangya Hospital of Central South University, Changsha, China; 8 School of Clinical Medicine, North Sichuan Medical College, Nanchong, China; 9 Department of Nuring, Affiliated Hospital of North Sichuan Medical Collge, Nanchong, China; 10 School of Public Affairs, Xiamen University, Siming District, 422 Siming South Road, Xiamen, China; 11 School of Public Health, Peking University, Beijing, China

**Correction: BMC Public Health 23, 1747 (2023)**

**https://doi.org/10.1186/s12889-023-16637-0**

The original publication of this article [1] contained the redundant affiliation 12 "Wuhan 430065, China". The original article has been updated to correct this error.
